# Supplementary material for: Impact of BCR-ABL1 Transcript Type on Response, Treatment-Free Remission Rate and Survival in Chronic Myeloid Leukemia Patients Treated with Imatinib
Source: J Clin Med. 2021 Jul 16;10(14):3146. doi: 10.3390/jcm10143146 (PMC8307111; doi:10.3390/jcm10143146)
Supplement: Supplementary file 1 [file jcm-10-03146-s001.zip › jcm-1280975-supplementary.pdf]

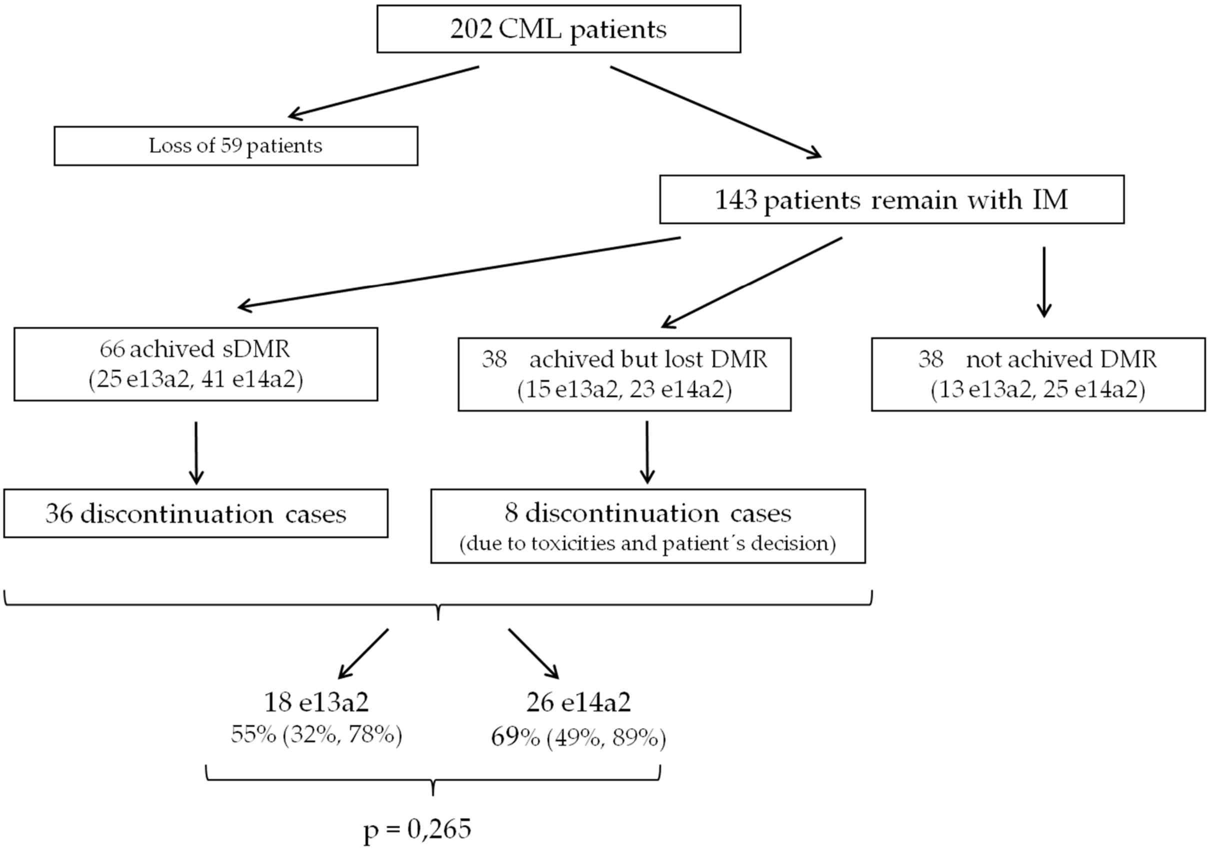


Figure S1. Diagram of CML cases in each analysis performed.

CML: Chronic Myeloid Leukemia; IM: Imatinib; sDMR: sustained deep molecular response; DMR: deep molecular response.
